# Reconstruction of Traumatic Open Extensor Loss With Achilles Calcaneus Allograft

**DOI:** 10.7759/cureus.84551

**Published:** 2025-05-21

**Authors:** Alex Mayers, Brian Campfield

**Affiliations:** 1 Orthopedic Trauma, Geisinger Health System, Scranton, USA

**Keywords:** achilles allograft reconstruction, extensor mechanism reconstruction, gustillo anderson 3b, knee extensor mechanism, proximal tibia fracture, trauma and orthopaedic

## Abstract

We report a 35-year-old male truck driver presenting after a motor vehicle rollover sustaining a 20 x 10 cm open grade 3B proximal tibia fracture with traumatic extensor mechanism loss undergoing acute extensor reconstruction and soft tissue coverage. After serial debridement bridged by local and intravenous (IV) antibiotics, definitive reconstruction with Achilles calcaneus allograft and medial-lateral gastrocnemius flap coverage was performed within 10 days. The patient achieved an excellent clinical outcome with <5-degree extensor lag, 100-degree knee flexion, and full quad strength. We demonstrate successful reconstruction of the traumatic extensor mechanism within the acute injury period.

## Introduction

Knee extensor mechanism injuries have an incidence ranging from 0.5% to 6% [1]. These injuries are usually seen in the sports or arthroplasty arena. Acute extensor mechanism injuries range from minor injuries with intact extensor apparatus to traumatic avulsions or disruptions requiring relatively simple surgical repair. Chronic conditions are represented after failed primary repairs or in multiply revised arthroplasty cases and often require more elaborate reconstructive surgeries with allograft or synthetic materials [2]. A rarer injury pattern is traumatic loss of significant extensor mechanism tissue. Treatment options in the traumatic loss of the extensor mechanism are similar to those of chronic deficiencies and include autograft or allograft reconstruction, whole patella allograft transplantation, and knee fusion.

## Case presentation

Case report

**Figure 1:**
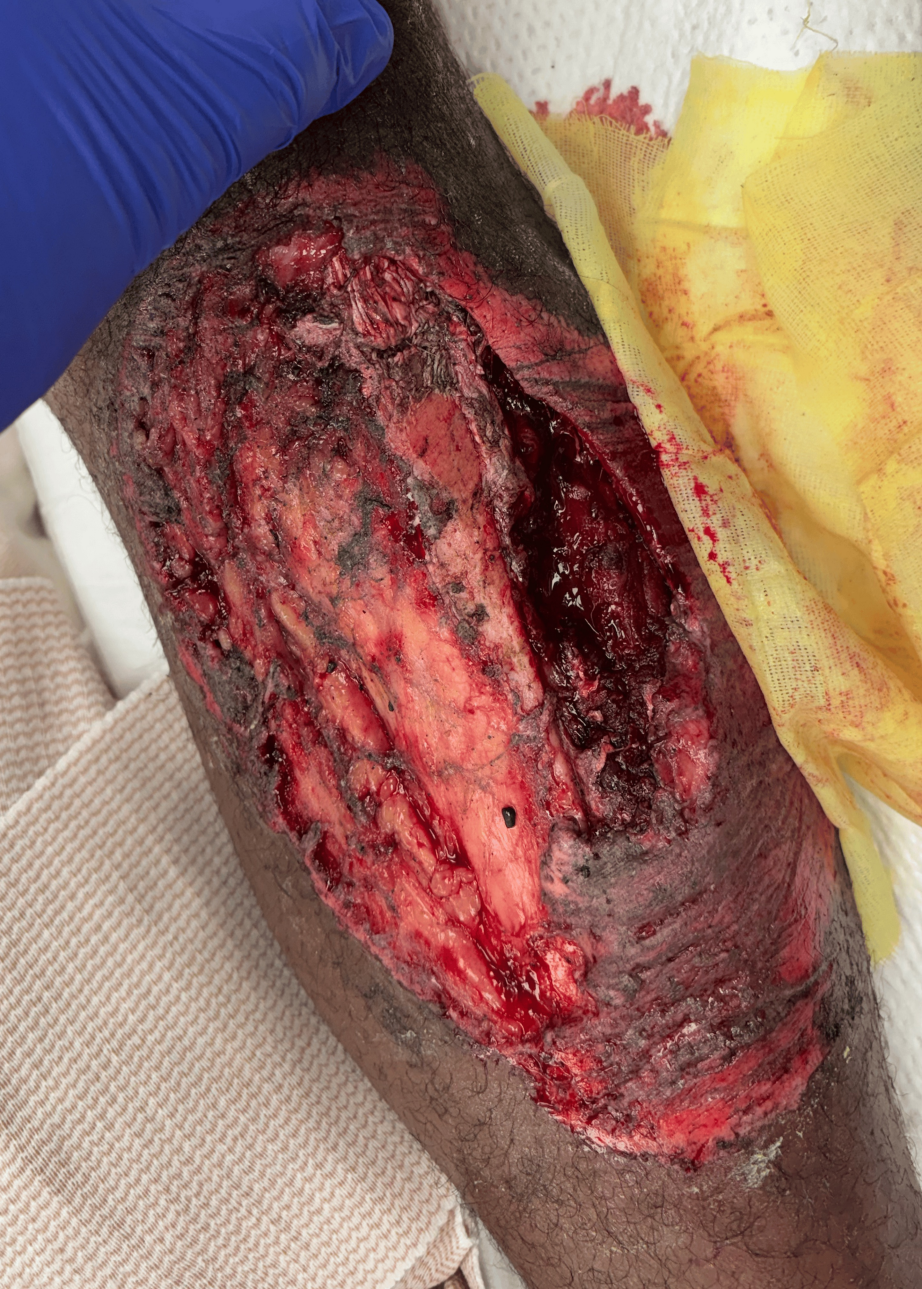
Grossly contaminated left anterior leg wound

We report a 35-year-old male truck driver presenting after a rollover motor vehicle collision, sustaining a left open grade 3B proximal tibia fracture with a traumatic, segmental extensor mechanism loss, thus requiring bony, tendinous, and soft tissue reconstruction. On arrival, he was hemodynamically stable with isolated musculoskeletal injuries. Secondary survey demonstrated a 20 x 10 cm grossly contaminated deep abrasion to the left anterior leg (Figure 1) with obliteration of the tibial tubercle, segmental loss of the patellar tendon, traumatic knee arthrotomy, left ankle dislocation, and left intraarticular distal radius fracture. Imaging of the right tibia demonstrated a coronal plane defect of the tibial tubercle and retained foreign bodies. Cefazolin and gentamycin antibiotics and tetanus prophylaxis were given. The patient was taken urgently to the operating room for exploration, debridement, and stabilization of these injuries. 

Consent 

**Figure 2:**
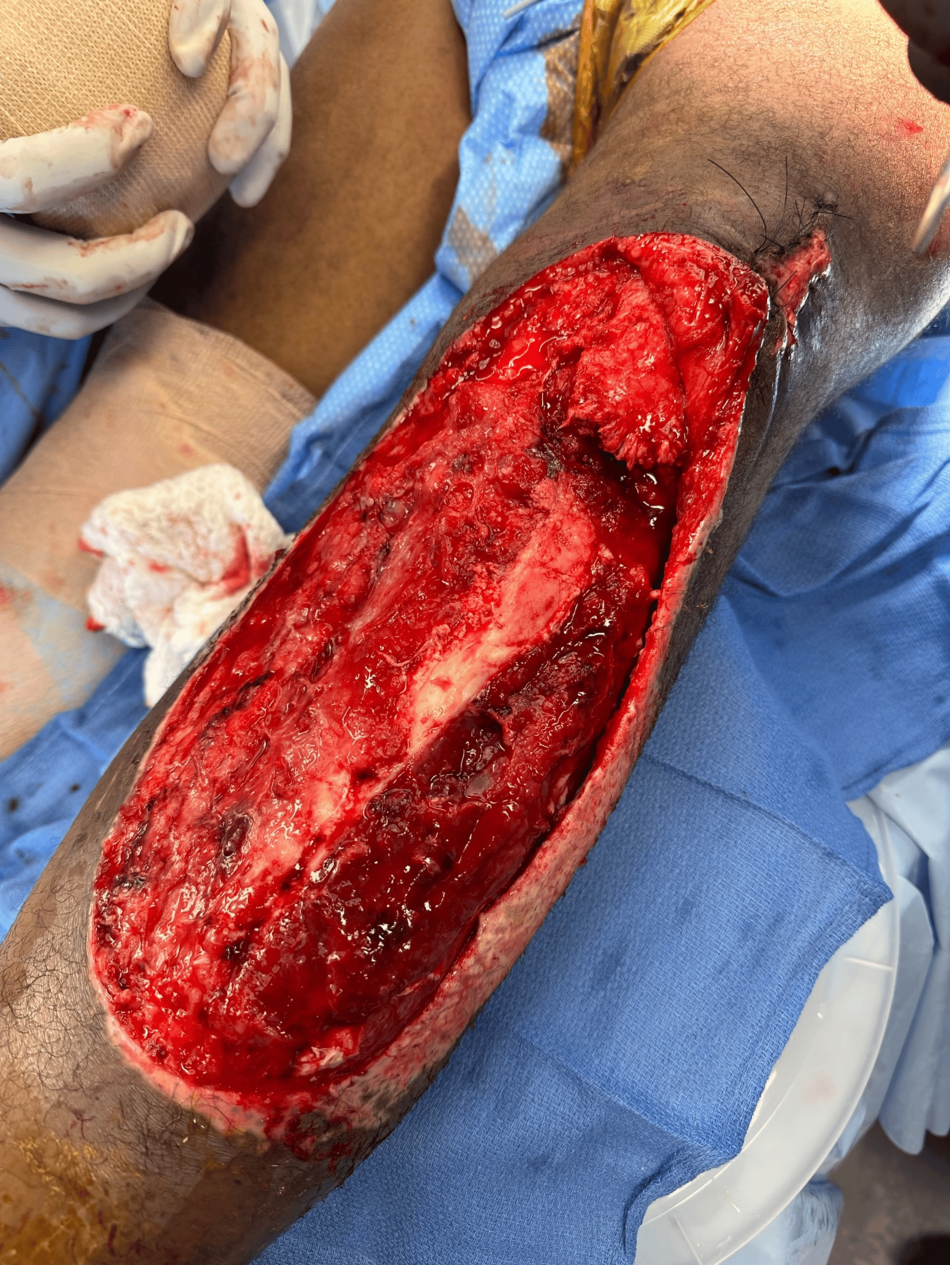
Left anterior leg wound postsurgical debridement

Formal consent was obtained from the patient for this report, including the use of radiographs and clinical images. The patient underwent serial debridement on hospital days (HD) 1, 3, 7, and 10). The initial debridement included exploration of a traumatic inferomedial parapatellar knee arthrotomy, which was free of gross contamination. The arthrotomy was extended and irrigated via a medial parapatellar approach and closed primarily. The large anterior lower leg wound was burdened with gravel and automotive glass depressed into exposed tibia as well as peripherally devitalized tissues. The proximal anterior and lateral compartments were exposed and devoid of overlying fascia. The periosteum of the medial proximal tibia was partially intact. The tibial tubercle was absent. There was avulsion and undermining of the lateral soft tissue envelope approximately 6 cm from the wound edge. Sharp manual debridement supplemented with the use of a hydrosurgery debridement system, combined with gentle and voluminous irrigation with normal saline, was effective in removing devitalized tissue and contaminants. The distal end of the patellar tendon was debrided to a healthy tendon, with a 6 cm defect at the insertion site. An antibiotic bead pouch was made with 3 grams of vancomycin, 3.6 grams of tobramycin, and 40 grams of methyl methacrylate cement formed into beads strung on a heavy suture and sealed with an adhesive membrane. Fluoroscopic evaluation of the ipsilateral ankle demonstrated tibiotalar instability without fracture. A spanning external fixator was placed across the ankle for the treatment of this recognized ankle dislocation. Under the same anesthetic, the left distal radius was fixed. The patient returned to the operating room for serial debridement on HD3 and HD7. The wound margins remained stable after the first debridement without progression of necrosis or infection, and the wound was deemed stable on HD7 with no further tissue loss, contaminants, or signs of infection (Figure 2).

Final Debridement and Reconstruction

On HD 10, the patient returned to the operating room for repeat debridement and irrigation of the wound, extensor mechanism reconstruction, application of knee-spanning external fixator incorporated with the previously placed ankle external fixation, and wound coverage. An Achilles tendon allograft with a calcaneus bone block was utilized for the reconstruction. A bone block allograft was utilized due to the loss of the tibial tubercle. 

**Figure 3:**
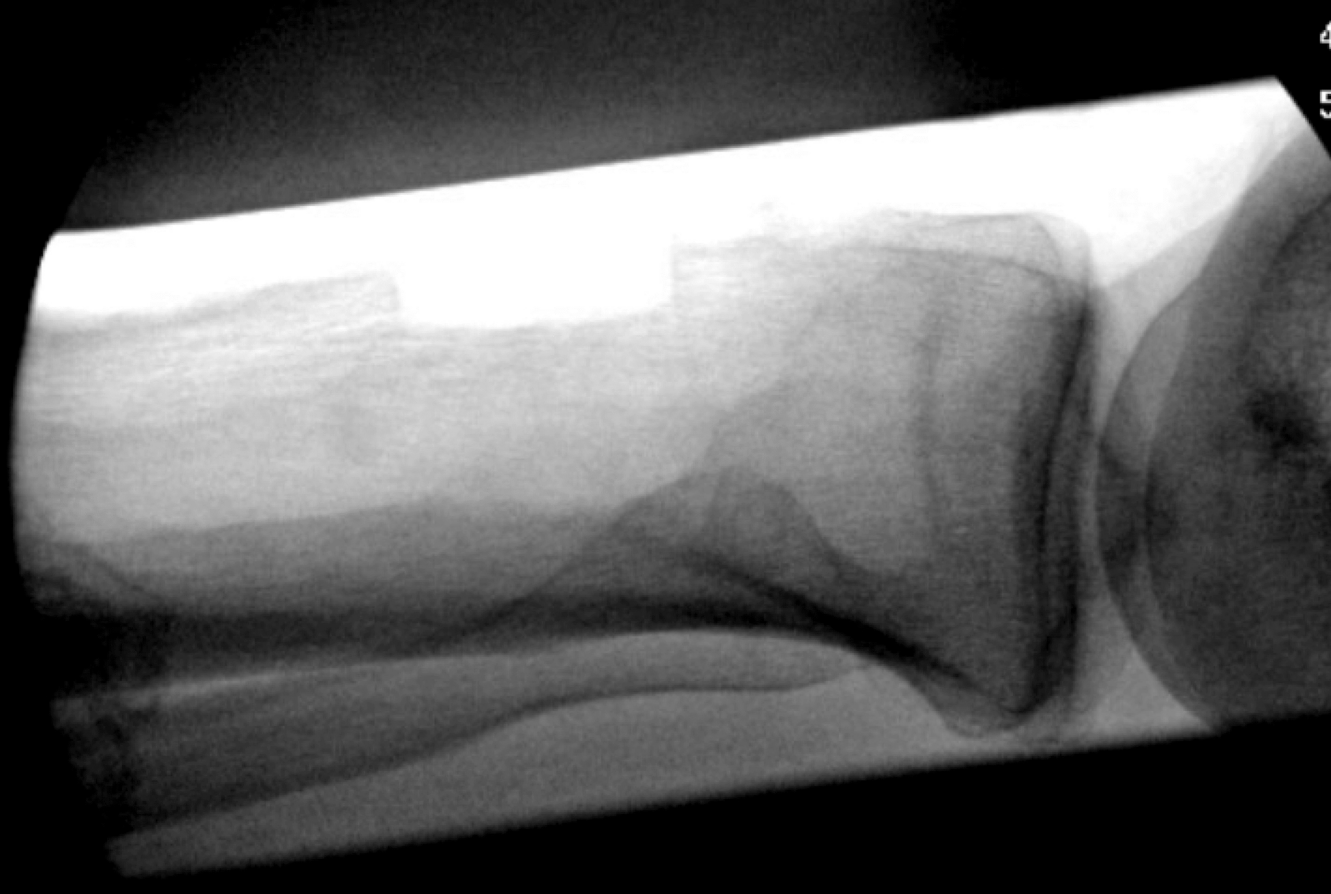
Trough osteotomy seen on lateral fluoroscopy

The bone block was measured and cut to fit the trough at the planned site of tibial tubercle reconstruction at the proximal tibia. The calcaneus-Achilles allograft was thawed. We trimmed the calcaneal bone block to occupy a space no larger than the dimensions of the tubercle defect. We also limited trimming such that Achilles insertion was not compromised. An appropriately sized trough was marked with a pen and Kirshner wires (K wires) in the proximal tibia. A microsagittal saw was utilized to create a trough osteotomy at the site of the tibial tubercle to fit our allograft bone block (Figure 3). 

**Figure 4:**
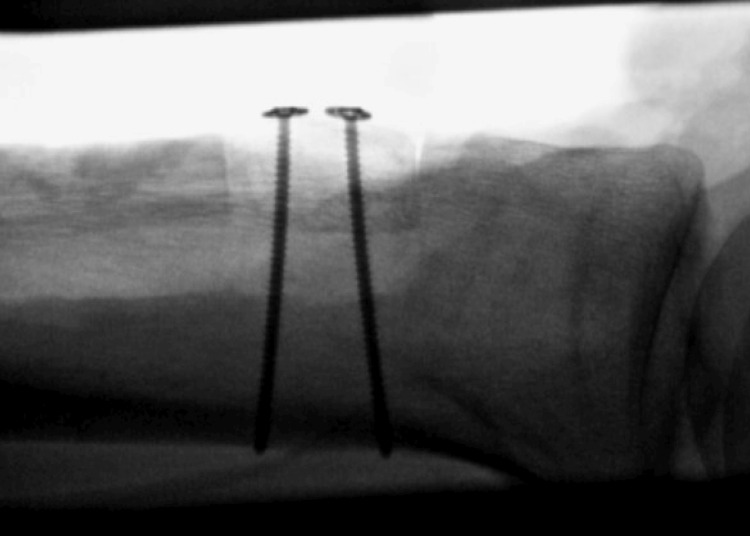
Lateral tibial fluoroscopy demonstrating bicortical screw fixation of calcanea bone block allograft into tibial trough

Bone marrow aspirate was percutaneously harvested from the distal lateral femur as it was immediately available within the prepped surgical field but remote from the site of open injury. The allograft bone was soaked in the bone marrow. The calcaneus allograft was gently impacted in place with a snug fit. This granted some inherent stability. The fit was satisfactory both clinically and fluoroscopically. Next, two 2.7 mm cortical screws with washers were placed as lag screws by technique anterior to posterior to fix the allograft to the proximal tibia with excellent compression (Figure 4).

Because orthotic-based knee immobilization was contraindicated due to planned flap reconstruction, the ankle-spanning external fixator was extended to span the knee to protect the extensor mechanism reconstruction. Two hydroxyapatite coated Schanz screws were placed bicortically in the femoral shaft and connected to the tibial pins with a carbon fiber bar laterally. 

**Figure 5:**
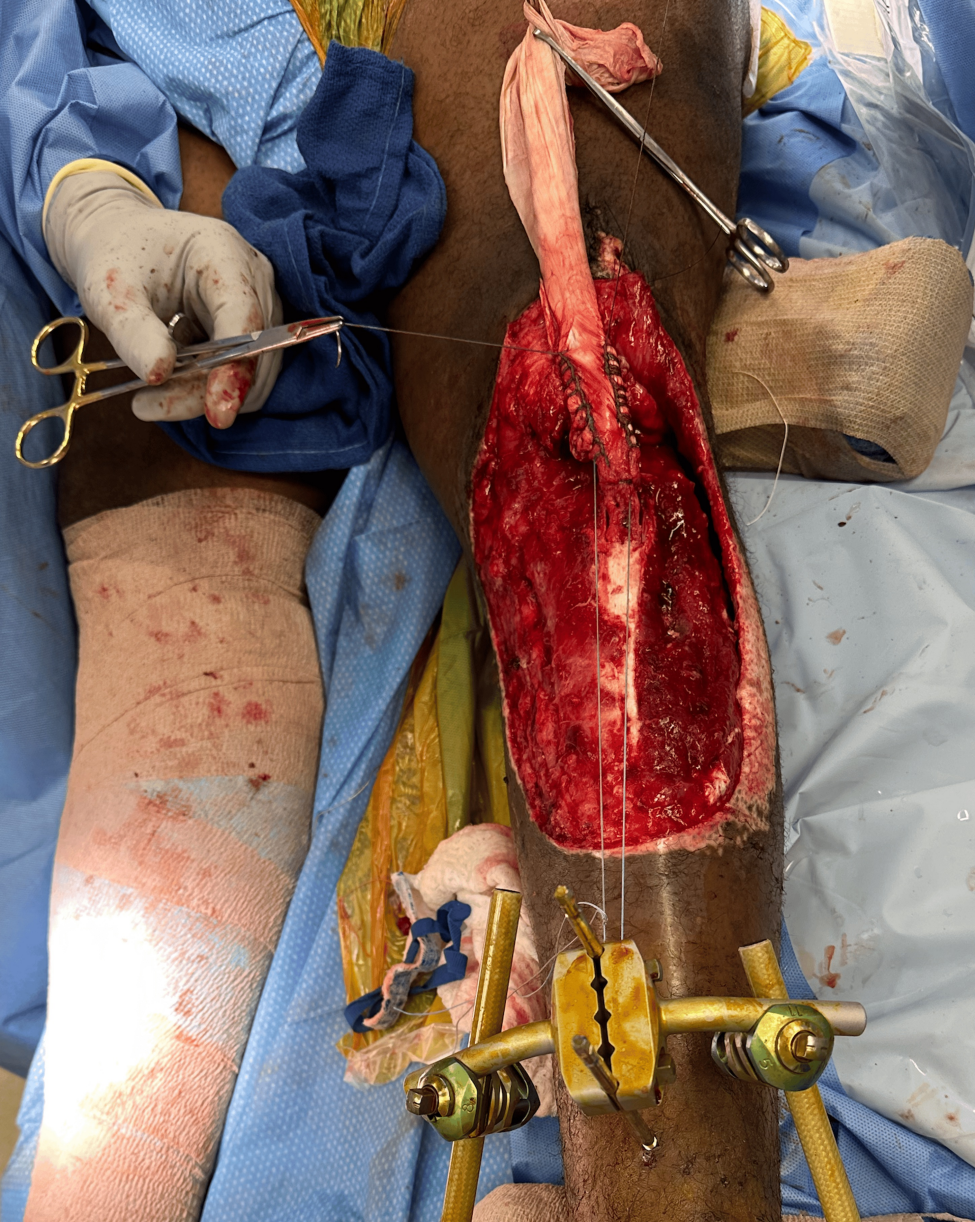
Intertendinous suture anchored to the tibial external fixator pins

**Figure 6:**
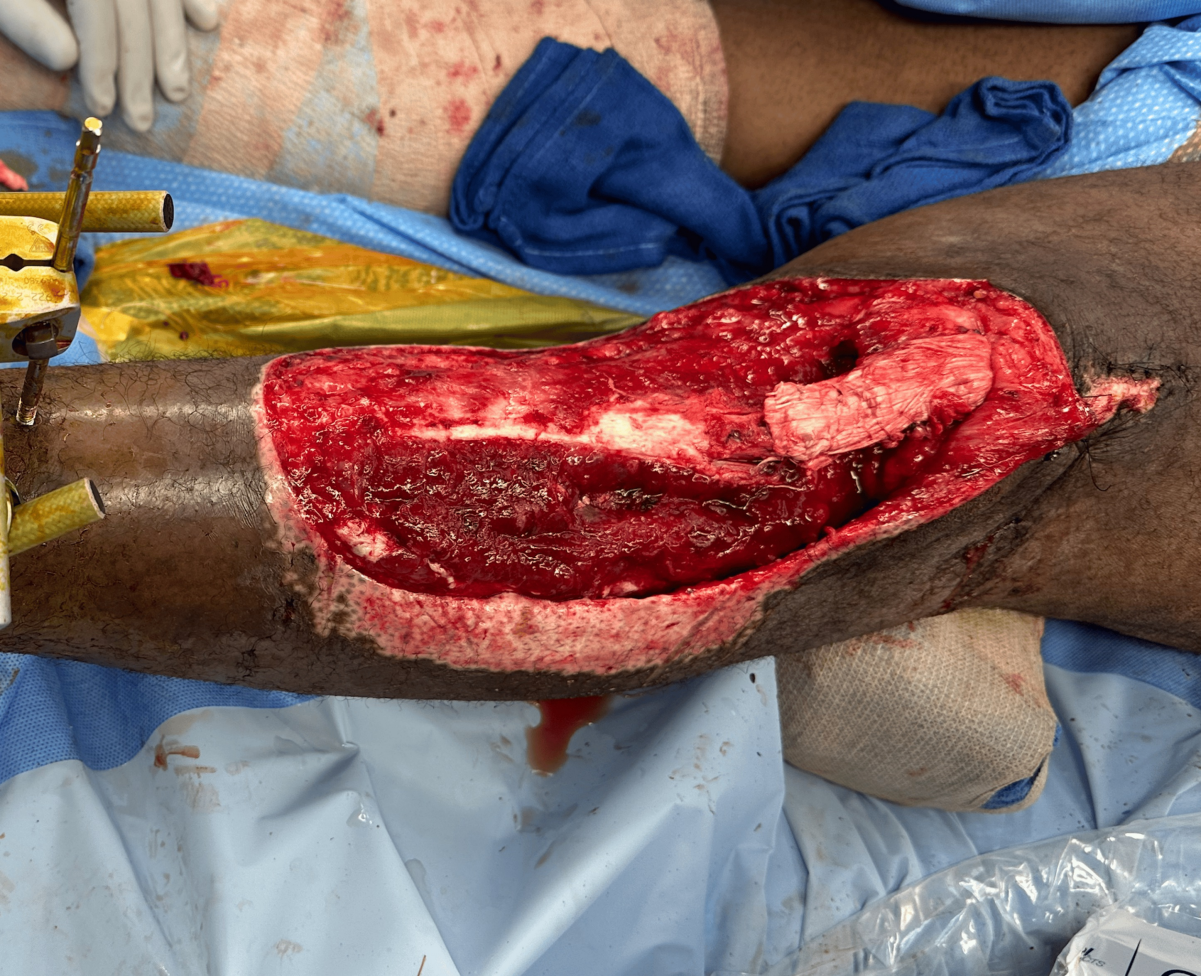
Completion of reconstruction with double-backed allograft

The stump of the patient's patellar tendon was tensioned deep to the Achilles allograft using an intertendinous suture anchored to the tibial external fixator pins (Figure 5). Patellar station was compared to the contralateral limb radiographically, then tensioned in the appropriate position. Heavy-duty braided suture was placed in a running locking configuration up the medial and lateral sides of the patellar tendon to incorporate the allograft to the stump of the native tendon. The allograft was then doubled back on itself down to the tibial tubercle, which was then sewn with a running 2-0 Vicryl suture to complete the extensor mechanism reconstruction (Figure 6). No osseous fixation was utilized proximally within the patella.

**Figure 7:**
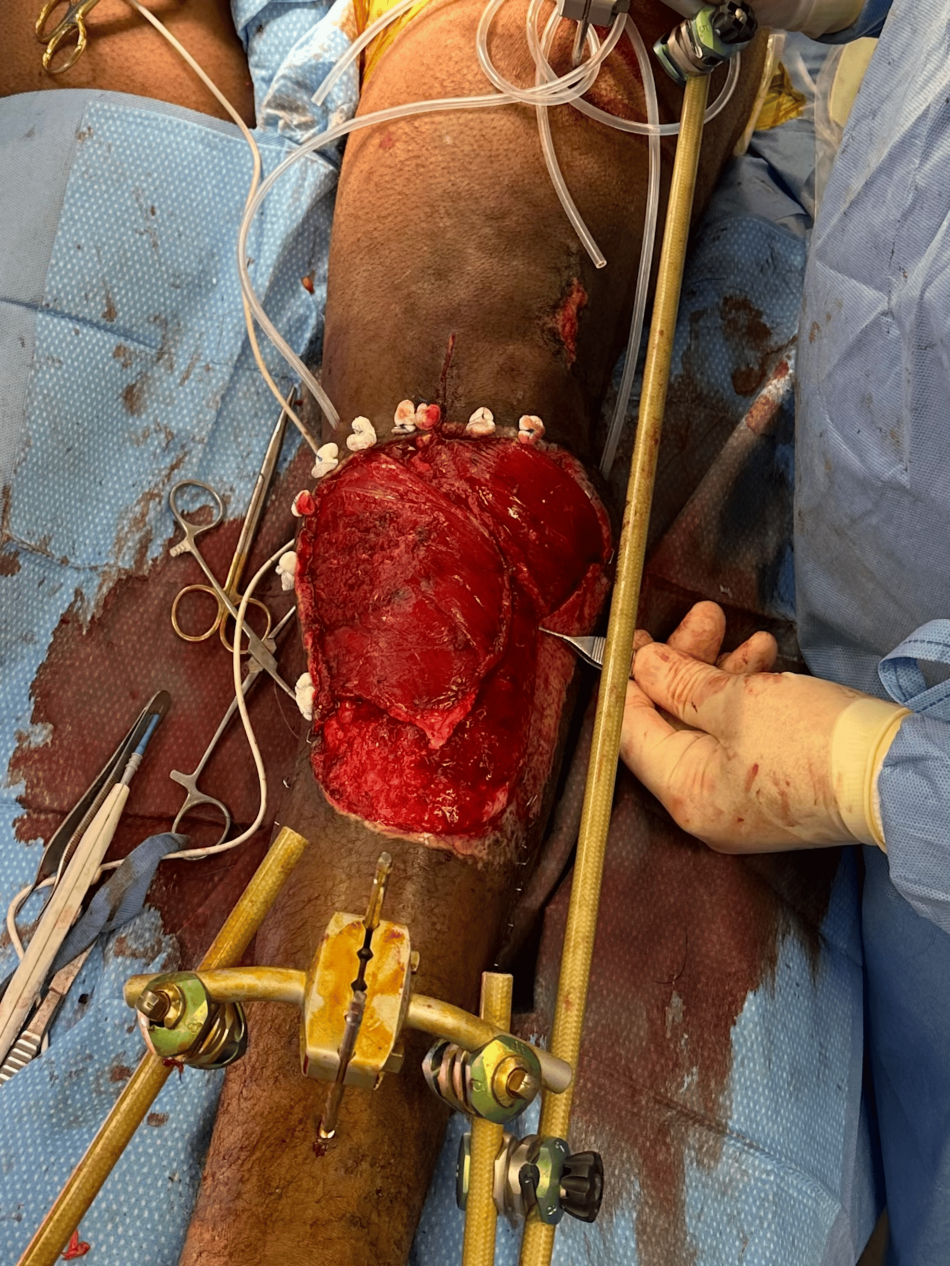
Gastrocnemius flap coverage and knee-spanning external fixation

Finally, the plastic surgery team performed the pedicured medial and lateral gastrocnemius flaps (Figure 7) and split thickness skin grafting for soft tissue coverage. Once the plastic surgery coverage was completed, a medial bar was added, and the external fixator was fully tightened.

Patient Outcome

**Figure 8:**
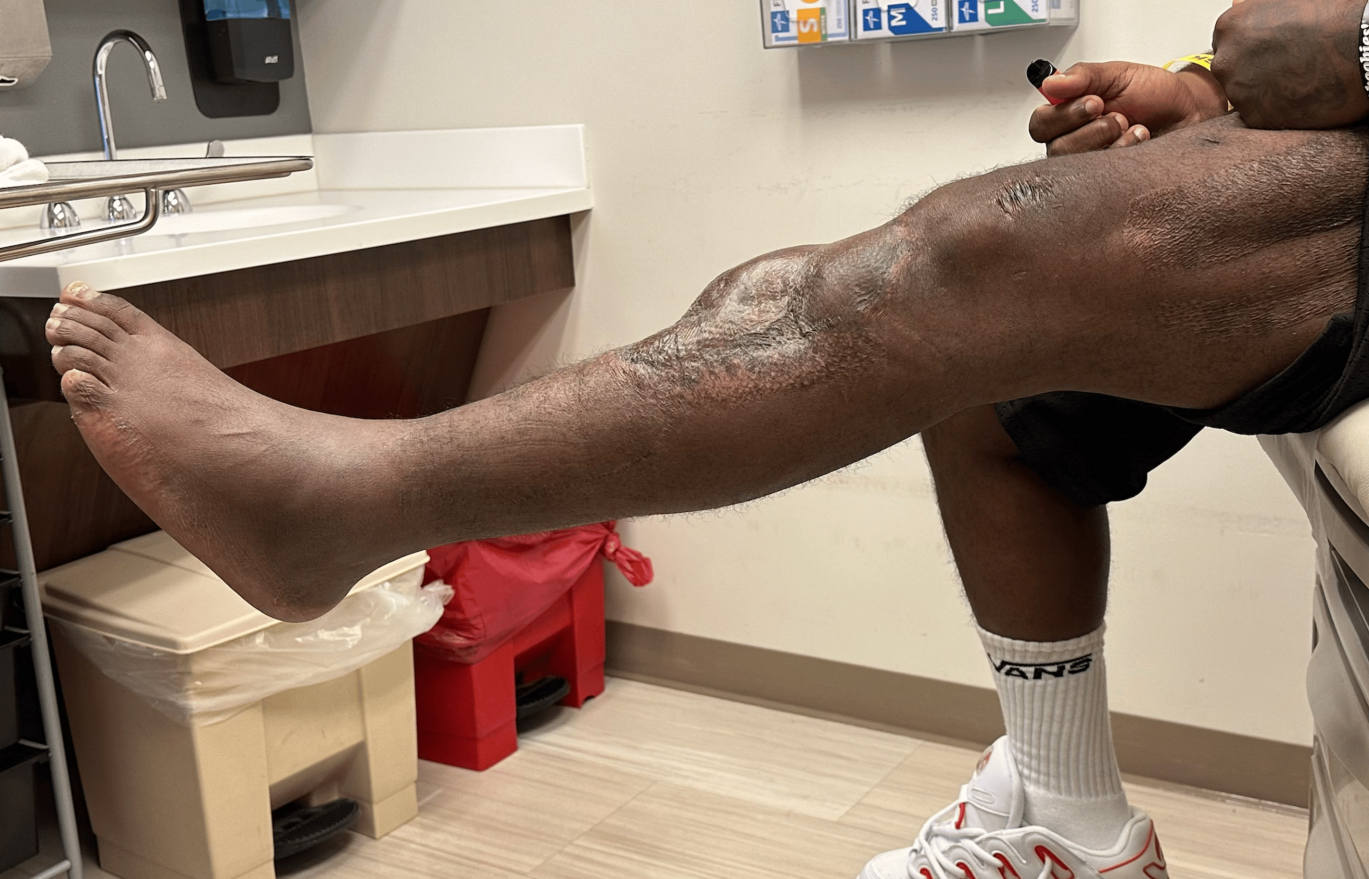
Eight month follow-up with a five-degree extensor lag

**Figure 9:**
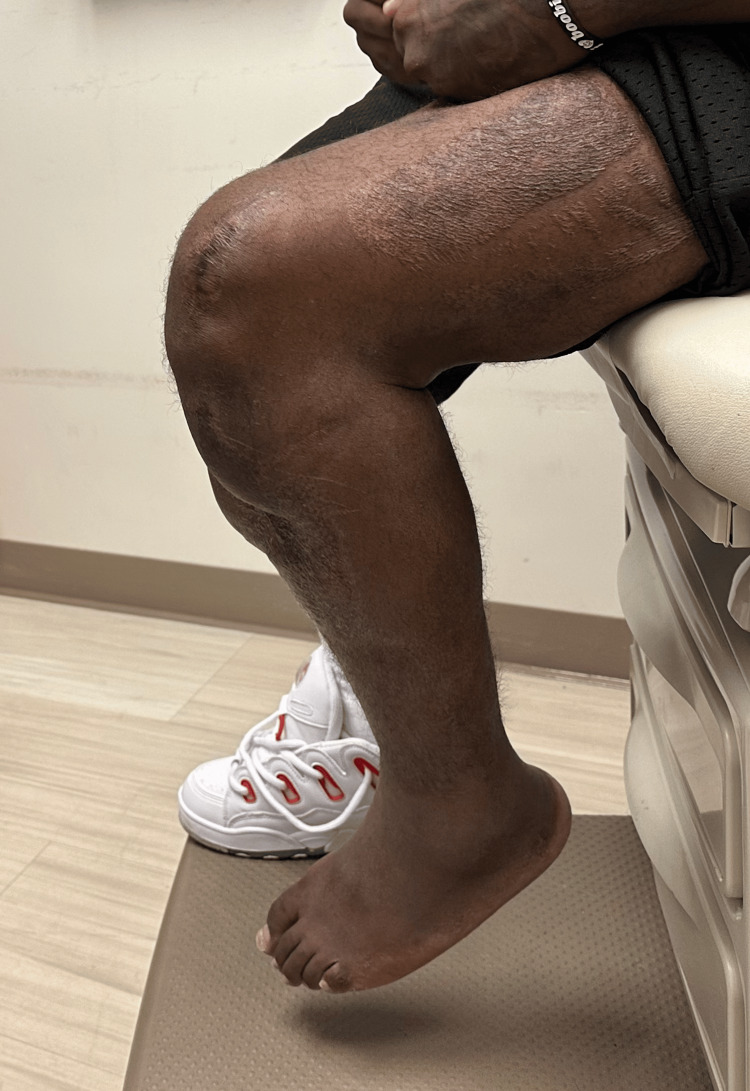
Eight-month follow-up with 100 degrees of flexion

**Figure 10:**
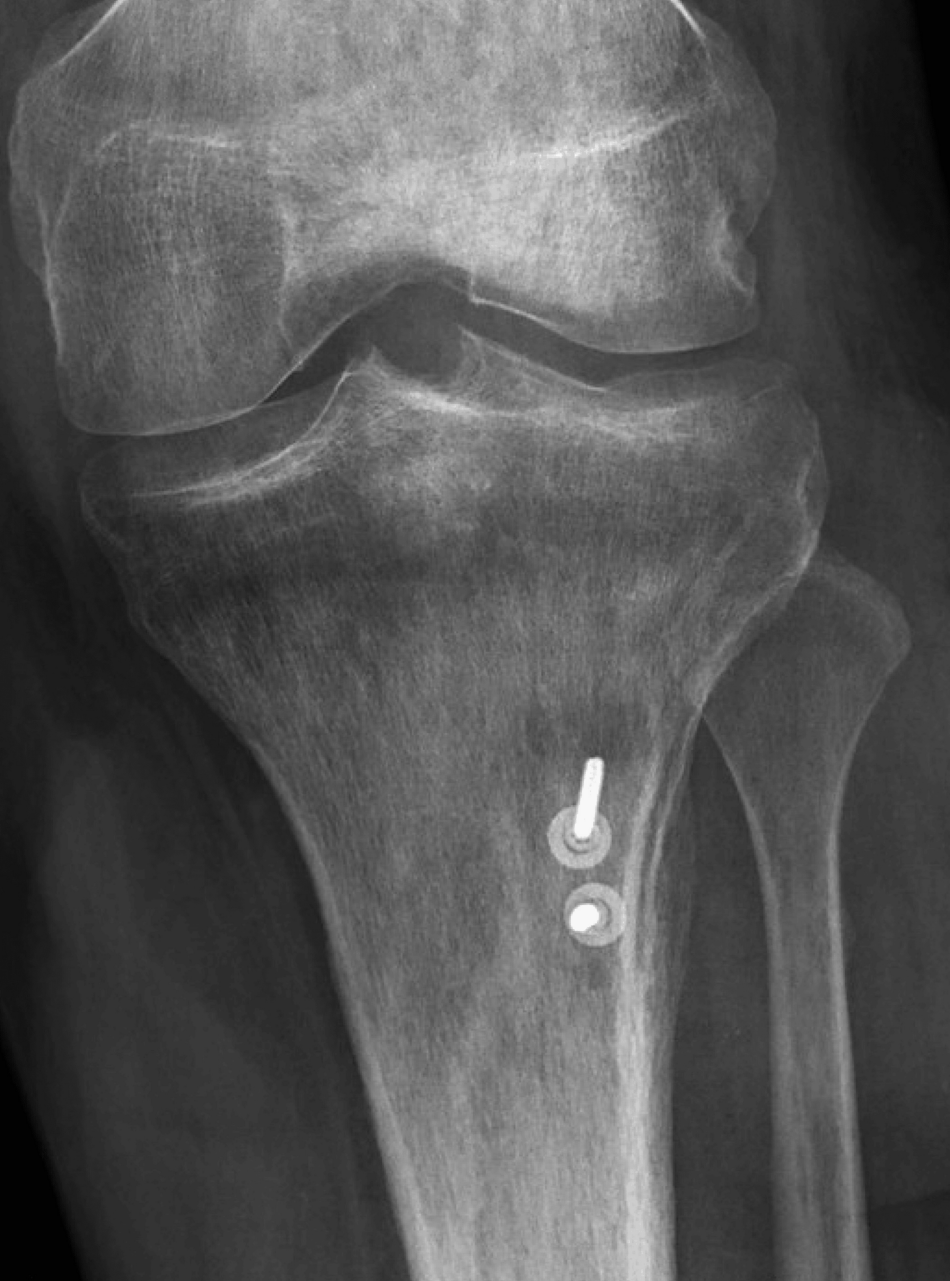
Eight-month AP radiograph of the left tibia demonstrating bony incorporation of calcaneus bone block allograft AP: anteroposterior

**Figure 11:**
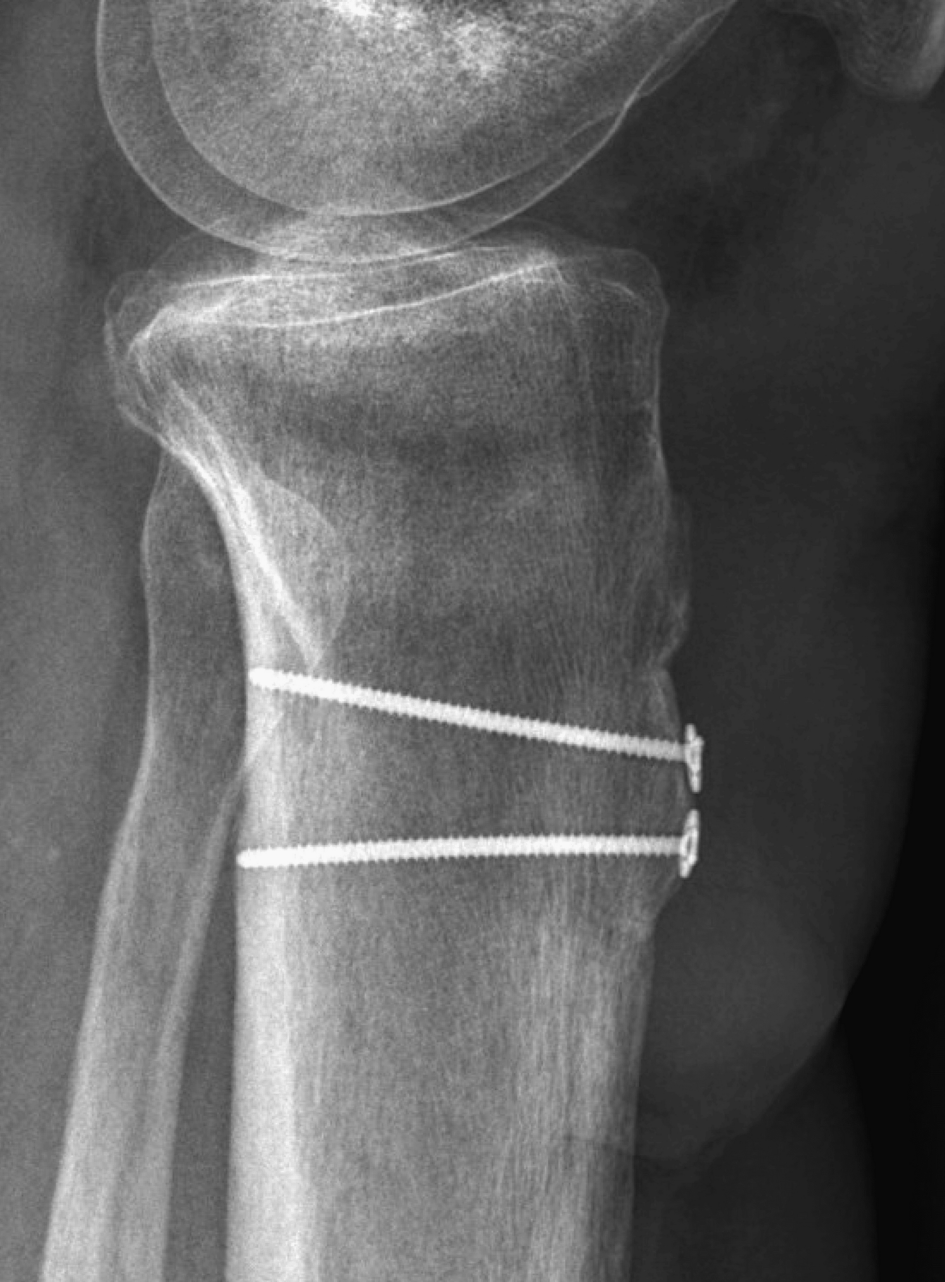
Eight-month lateral radiograph of the left tibia demonstrating bony incorporation of calcaneus bone block allograft

The external fixation was maintained for eight weeks to allow healing of the reconstruction and protect the soft tissue coverage. After eight weeks, the external fixator was removed in the clinic, and the patient was transitioned to a hinged knee brace unlocked 0-60 with a walking cam boot. He was made weight bearing as tolerated with a walker and allowed to advance 10 degrees of knee flexion weekly with therapy. The patient was seen at three months postoperatively, at which point all surgical wounds were healed, including complete incorporation of the skin graft. He demonstrated a 10-degree extensor lag with full passive extension and was able to flex to 60 degrees. At the most recent in-person follow-up, eight months postreconstruction, he demonstrated no evidence of infection, five-degree extensor lag (Figure 8), 100 degrees of flexion (Figure 9), and 4+/5 quadriceps strength, which continues to improve. He is bearing weight as tolerated in regular lace-up shoes and sometimes utilizes a cane for distances greater than a few hundred yards. He notices weakness with plantar flexion, as may be expected with an extensive gastrocnemius flap. Radiographically, the allograft bone is well incorporated (Figures 10, 11). He has not returned to work as he was terminated from his previous position and has not chosen to seek new employment at this time. At 12 months postsurgery, the patient is living out of state with no intention of returning for follow-up due to financial constraints. He continues to be unemployed and is seeking permanent disability; however, he states he is walking independently with mild chronic knee pain, full extension, and approximately 100 degrees of knee flexion.

## Discussion

After an extensive literature review, we found no reports or series on the reconstruction of extensor mechanisms after traumatic open segmental tissue loss and contamination of this caliber with the use of Achilles-calcaneal allograft. We did identify one case of extensor mechanism reconstruction after traumatic tissue loss by Raschke et al.; however, the reconstruction methods differed significantly [1].

Extensor mechanism reconstruction is described in the context of several techniques, including biceps autograft by Peyers et al., dacron or marlex synthetic reconstruction by Silva et al. [3,4]. Reconstruction using Achilles tendon allograft with Achilles bone block is a described technique within the sports literature. Strother et al. manage first-time ruptures with significant tendinosis or moderate tissue loss with quadrupled semitendinosus tendon autograft augmentation, reserving the Achilles allograft reconstruction technique after failed prior extensor mechanism repair or reconstruction and poor tissue quality. They retrospectively identified 22 patellar and 21 quadricep tendon reconstructions, 13 of which underwent Achilles reconstruction. At the last follow-up >1 year, four cases had extensor lag >5 degrees, no cases required additional surgery, and all cases were able to achieve >90 degrees of knee flexion [5]. Their technique differs from ours in that sutures are passed through patellar drill tunnels. Karas et al. noted a 33% incidence of persistent extensor lag after extensor mechanism allograft reconstruction [6]. We specifically chose an allograft with a calcaneal bone block due to the bony tubercle deficit in this patient. We note a mild five-degree but not functionally limiting extensor lag in this case report.

Our technique differed in that the Achilles tendon was incorporated into the remaining patellar tendon stump without transosseous augmentation. Additional transosseous fixation could have been employed; however, it was deemed unnecessary in this case due to the robust remaining tendon stump. The extensor reconstruction portion of the procedure could have been staged after flap coverage; however, given the need to raise and work around the flap as well as the aggressive nature of the debridement, the wound was felt to be ready for definitive reconstruction at the time of coverage. Jentzsch et al. note that extensor lag is associated with gastrocnemius flap coverage; however, we consider it to be a consequence of injury or disease severity and not a direct influence of flap coverage [7]. In this case, reproaching a flap may lead to additional scarring and further extensor lag or reduced range of motion. Jentzsch et al. also note that patella alta is associated with extensor lag, greater flexion, and worse functional scores but stabilized after two years. For this reason, we would prefer to follow our patient peripherally for two years; however, financial and social constraints limit this patient's follow-up to the present date [7].

## Conclusions

We provided a case report of knee extensor mechanism reconstruction with Achilles calcaneal allograft in the acute trauma period. This type of reconstruction is an effective procedure for restoring the extensor apparatus in the setting of traumatic extensor loss. The calcanea Achilles allograft is our preferred choice over other grafts. It evades the morbidity of autograft options, does not require bone-tendon healing at the insertion site, and has demonstrated to us radiographic and clinical incorporation. There is a paucity of literature and guidance in this specific scenario. A case series would be the next logical step. This type of injury requires a multidisciplinary approach, including the expertise of orthopedics, plastics, physiotherapy, and rehabilitation. Given that these patients have difficulty with extensor lag and range of motion, improvement in reconstruction techniques will remain an area of interest.
